# Revealing the Acute Effects of Dietary Components on Mood and Cognition: The Role of Autonomic Nervous System Responses

**DOI:** 10.3390/brainsci13081177

**Published:** 2023-08-08

**Authors:** Sélima Zahar, Evelina De Longis, Julie Hudry

**Affiliations:** 1 Brain Health Department, Nestlé Institute of Health Sciences, Nestlé Research, Société des Produits Nestlé S.A., 1000 Lausanne, Switzerland; evelina.delongis@rd.nestle.com (E.D.L.); julie.hudry@rdls.nestle.com (J.H.); 2 Center for Neuroprosthetics, Neuro-X Institute, École Polytechnique Fédérale De Lausanne (EPFL), 1202 Geneva, Switzerland

**Keywords:** dietary components, autonomic nervous system, stress, alertness, cognition, digital technologies, personalized nutrition

## Abstract

A growing body of literature suggests dietary components can support mood and cognitive function through the impact of their bioactive or sensorial properties on neural pathways. Of interest, objective measures of the autonomic nervous system—such as those regulating bodily functions related to heartbeat and sweating—can be used to assess the acute effects of dietary components on mood and cognitive function. Technological advancements in the development of portable and wearable devices have made it possible to collect autonomic responses in real-world settings, creating an opportunity to study how the intake of dietary components impacts mood and cognitive function at an individual level, day-to-day. In this paper, we aimed to review the use of autonomic nervous system responses such as heart rate or skin galvanic response to investigate the acute effects of dietary components on mood and cognitive performance in healthy adult populations. In addition to examining the existing methodologies, we also propose new state-of-the-art techniques that use autonomic nervous system responses to detect changes in proxy patterns for the automatic detection of stress, alertness, and cognitive performance. These methodologies have potential applications for home-based nutrition interventions and personalized nutrition, enabling individuals to recognize the specific dietary components that impact their mental and cognitive health and tailor their nutrition accordingly.

## 1. Introduction

In our rapidly changing world, various factors can affect our body and environment, leading to changes in mood and cognitive performance. Upon detecting a factor or a stimulus, the body can enter a state of alertness [1] and its homeostasis can be disrupted, leading to a state of stress [2]. To maintain the body’s homeostasis or prepare the body for action, the autonomic nervous system (ANS) initiates a cascade of physiological signals. These signals guide modifications in behaviour and cognitive function to address the new demands associated with the stimulus [3,4]. Certain dietary components with bioactive mechanisms (e.g., caffeine) or sensorial properties (e.g., peppermint) can act on the neural circuitry involved in mood and cognitive function, potentially helping individuals better react to novel situations and demands [5,6].

Although several nutritional interventions have evaluated the acute effects of dietary components on stress, alertness, and cognitive performance using standardized and/or valid approaches, these traditional methods have limitations that can lead to biased outcomes [7,8,9]. These methods, including subjective assessments and cognitive tasks, require repeated measurements for accuracy and experimental manipulation, potentially leading to habituation and cognitive bias [7,8]. Additionally, subjective assessments can lead to biased outcomes as they must be reported at time points determined before the experiment [8]. Lastly, subjective ratings can introduce subjective bias into the experiment’s results [9].

Recently, the use of ANS responses has emerged as a reliable and objective approach for assessing the acute effects of dietary components on stress, alertness, and cognitive performance [8,9]. By responding to changes in the effort associated with cognitive function as well as changes in arousal and valence linked to mood, ANS responses have the potential to provide reliable measures for evaluating the effects of dietary components [10,11]. The sympathetic nervous system (SNS), one of the two sub-branches of the ANS, is typically associated with a state of arousal that underlies high levels of stress or alertness [12]. On the other hand, the activation of the parasympathetic nervous system (PNS), the other sub-branch of the ANS, is linked to physical well-being and a state of positive valence, which can underly a low level of stress [13]. Additionally, the prefrontal cortex may indirectly regulate ANS activity to maintain the effort required for cognitive function to meet the demands of everyday living [14]. Hence, the SNS is activated during cognitive effort, while the PNS may help regulate this effort and facilitate recovery. The SNS mediates physiological changes such as pupil dilation, increased heart rate (HR), peripheral vasoconstriction, and enhanced sweat gland secretion. PNS activation leads to antagonist responses, particularly a decrease in HR, resulting in enhanced heart rate variability (HRV) [10,11].

Several intervention studies have attempted to demonstrate the acute effect of dietary components on stress, alertness, and cognitive performance by recording ANS responses in complement to cognitive tasks and subjective ratings that are standardized and/or valid [15,16,17,18,19,20,21,22,23,24,25,26,27,28]. In these studies, ANS responses are typically assessed using laboratory devices such as electrocardiograms (ECGs) to measure HR [29], skin electrodes to track the increase in skin galvanic response (GSR) resulting from sweat gland secretion [30], eye trackers to quantify pupil dilation [31], or thermistors to measure changes in skin temperature [32]. The measurement of ANS responses in conjunction with cognitive tasks and subjective assessments has allowed the demonstration of the acute effects of dietary components on mood states and cognitive function in several studies [19,20,21,22,26,33].

This paper aims to provide a narrative review of the use of ANS responses for the investigation of acute nutritional intervention’s effect on cognitive performance, stress, and alertness in healthy adult populations. The corpus of reviewed studies consists of acute nutritional interventions based on single-day assessments. Only original research articles written in the English language were considered. The review ends with a discussion on the potential use of ANS proxy patterns for detecting changes in mood and cognitive function, and their application in home-based and personalized nutritional interventions aimed at improving the effectiveness of nutrition research.

## 2. Dietary Components to Relieve Stress Acutely

Studies have shown the potential usefulness of ANS responses as a means by which to assess the acute effect of dietary components on stress (Table 1). Among natural sources of amino acids, Tryptophan and L-theanine have received particular attention due to their ability to modulate stress by targeting neural pathways responsible for regulating mood [5,34]. A recent study by Zahar S, Schneider N, Makwana A, Chapman S, Corthesy J, Amico M and Hudry J [35] suggested that an egg protein hydrolysate food matrix incorporating a dietary source of the amino acid Tryptophan at a 1 g dose can reduce ANS response to acute stress induced by cognitively demanding tasks. This was reflected by an increase in HRV in comparison with a placebo. Similarly, the findings of another study examining the effects of L-theanine intervention (oral administration of a 200 mg dose diluted in water) 20 min post-absorption demonstrated a reduction in autonomic response to stress induced by a mental arithmetic task, as evidenced by lower HR compared with a placebo [36]. Additionally, sensory properties can promote stress relief by directly stimulating the senses [6]. A study by Jung D-J, Cha J-Y, Kim S-E, Ko I-G and Jee Y-S [37] revealed that inhaling Ylang-Ylang essential oil extracted from flowers of the tropical tree Cananga odorata for a duration of 20 min effectively reduces the cardio-autonomic responses associated with stress. This was evidenced by a noticeable decrease in both HR and blood pressure in healthy male participants.

Research attempting to mediate stress via dietary intervention has yielded mixed findings from ANS outcome measurements (Table 1). Despite strong indication that the herbal component “green oat” can reduce stress [39], this was not supported by results from stress-related measurements conducted by Kennedy DO, Bonnlander B, Lang SC, Pischel I, Forster J, Khan J, Jackson PA and Wightman EL [16]. Indeed, the findings of this study did not show a reduction in GSR in an experimental procedure of multiple cognitive tasks with a targeted stress effect. Similarly, the reduction in subjective stress observed by Boyle NB, Billington J, Lawton C, Quadt F and Dye L [38] following the intake of a combination of magnesium (150 mg elemental), green tea (125 mg containing 40% L-Theanine), rhodiola extract (222 mg), and B vitamins (0.7, 0.1, and 0.00125 mg of vitamins $B_{6}$, $B_{9}$ and $B_{12}$, respectively) was not associated with a reduction in autonomic arousal. This disconnection between ANS and subjective measures of stress was also noticed in the Tryptophan intervention conducted by Zahar S, Schneider N, Makwana A, Chapman S, Corthesy J, Amico M and Hudry J [35].

Finally, it is important to note that the intake of certain dietary components can be detrimental to the mood by enhancing stress, especially if the component is consumed in high doses. By examining HRV during the two hours following consumption of glucose, fructose, and a combination of the two, Eckstein et al. [17] noticed a trend towards a higher autonomic response to stress compared with a placebo. A close link between blood glucose levels and the ANS response was also found in this study, suggesting overall a quick and increased sympathetic reaction induced by carbohydrate consumption.

## 3. Dietary Components That Acutely Enhance Alertness

Evidence shows the potential of ANS responses to mirror changes in alertness following the intake of a dietary component (Table 2). This is particularly supported by caffeine studies. Caffeine’s well-known effect on alertness is mediated through the antagonism of adenosine receptors which, in turn, leads to a stimulated release of neurotransmitters such as noradrenaline, dopamine, and acetylcholine [40]. Following a typical absorption period of 30 min, caffeine administration has been associated with sympathetic activation together with an increase in subjective alertness and/or a decrease in reaction time to a sustained task (RT) as another measure of alertness [19,20,21,22,26]. Similarly, Redondo B, Vera J, Carreño--Rodríguez C, Molina-Romero R and Jiménez R [19] showed increased pupil dilation and subjective rating of alertness 30 min following the intake of a caffeine beverage (caffeine doses ranged from 200 mg to 340 mg adjusted by the subject’s weight) compared with a placebo. This supports previous observations that heightened sympathetic activity, which can be induced by caffeine, causes a contraction of the iris dilator muscle, leading to pupil dilation [41]. Similarly, evidence of sympathetic activation following consumption of caffeine (dose ranging from 37.5 to 150 mg incorporated in a beverage) was demonstrated relative to a placebo, through the increase in GSR after an absorption period of 10–30 min in a study by Quinlan PT, Lane J, Moore KL, Aspen J, Rycroft JA and O’Brien DC [26]. Sensory properties of dietary components might also play a key role in the enhancement of alertness by triggering an immediate activation of nervous circuits involved in mood regulation e.g., trigeminal pathways [6]. Such immediate effects on subjective and cognitive measures of alertness were recently observed in a study testing a carbonated caffeinated beverage compared with caffeine alone and a control beverage [42]. Similar findings were suggested by the increase in GSR following the ingestion of hot water as another beverage matrix for coffee [26]. On this same note, the effects of a deep inhalation of essential oil scents consisting of peppermint and rosemary showed an increase in HRV together with a rise in RT [33].

Acute nutritional interventions targeting effects on alertness with ANS responses as outcome measures have also resulted in mixed findings (Table 2). While the hypothesis that ‘caffeine enhances alertness’ was supported by the increase in GSR, it was also contradicted by the trend towards a decrease in HR and an increase in blood pressure in the study conducted by Quinlan PT, Lane J, Moore KL, Aspen J, Rycroft JA and O’Brien DC [26]. This pattern might reflect a predominance of the baroreflex mechanisms being activated by caffeine [40]. The effects of caffeine can also depend on the composition of the matrix through which it is administered [43]. In the study by Bichler A, Swenson A and Harris M [44], the ingestion of a mix of caffeine and taurine (incorporated in a pill at serving doses of 100 mg and 1000 mg, respectively) was followed by a decrease in HR 45 min after intake. It may be that caffeine induced a baroreflex-mediated response, reflected by a decrease in blood pressure, potentially facilitated by taurine, as an amino acid with a vasoactive property. Numerous studies have sought to examine the effects of drinks with differing sensory properties and containing stimulating ingredients such as caffeine, taurine, and glucose on outcomes linked to alertness. Whether the effect of energy drinks on alertness is the outcome of a synergy between the active compounds and the sensory property of the matrix remains a question for research [45,46]. The absence of an immediate post-treatment effect on reaction time task performance in the acute testing of an energy drink in the study conducted by Smit HJ and Rogers PJ [46] suggests that the sensory property of the beverage did not enhance alertness. However, in this study, both the sensory attributes and the bioactive constituents of the drink may have led to a synergistic effect that resulted in enhanced alertness given that RT improvements were observed 80 min after treatment.

## 4. Dietary Components to Improve Cognitive Performance Acutely

Scientific literature provides evidence supporting the utilization of ANS responses in enhancing our comprehension of the acute impact of dietary components on cognitive performance (Table 3). The improvement in cognitive performance can be the result of the bioactive or sensorial properties of the dietary component. The ability to maintain cognitive effort could also be favorized by enhanced alertness or stress reduction. Indeed, synergistic effects on mood states and cognitive performance were observed in several caffeine intervention studies [23,24,47]. Evidence of caffeine’s impact on ANS activity suggests an increase in HRV and pupillary dilation after the consumption of the component [48]. Improvement in cognitive performance can also be acutely induced with non-nutritional interventions. By triggering a higher cerebral activity through mastication, chewing gum might enhance the delivery of oxygen and glucose to neural regions involved in cognition, leading to better cognitive performance [49].

Studies investigating the effects of dietary components on cognitive performance using ANS responses as outcome measures have resulted in mixed findings (Table 3). While there appears to be an association between caffeine consumption and cognitive function, several factors may influence this relationship, including the timing of consumption, dosage, experimental conditions, expectancy bias, population demographics, and habitual intake of caffeine [53,54,55]. In the study conducted by Pomportes and colleagues [52], a decrease in parasympathetic modulation was observed following caffeine (100 mg) ingestion. In the same study, the ingestion of a multi-vitamin-mineral preparation supplemented with 300 mg guarana led to a significant improvement in decisional cognitive performance and stability in parasympathetic modulation. Relatedly, the constituents of dietary components can synergistically impact cognitive performance, as demonstrated in the study by Scholey AB and Kennedy DO [45]. Their research found that while neither herbal extract flavoring nor caffeine alone improved cognitive performance, an energy drink containing all these constituents led to better memory and attention performance compared with a placebo. Furthermore, despite the finding that gum chewing improved alertness during the workday in the study conducted by Allen AP and Smith AP [50], it did not show any effect on HR. Conversely, Allen AP, Jacob TJ and Smith AP [51] noticed that gum chewing was associated with a decrease in HR, possibly reflecting an attenuation of sympathetic arousal. This was also associated with reductions in response rate in a vigilance task. Altogether, these effects supported a decrease in arousal. However, when participants completed the vigilance task, the chewing gum intervention showed a coordinated increase in HR with a decrease in task reaction, suggesting a suboptimal rate of arousal. The results from the above-mentioned studies suggest that gum chewing induces variations in arousal that relate to changes in cognitive performance according to an inverted U-shaped function [56]. However, the experimental ECG setup used by Allen AP, Jacob TJ and Smith AP [51] can be discussed as it may have biased the rating of alertness, which was quite high at baseline [53,54,55].

## 5. Discussion

In this paper, we aimed to present a review of the use of ANS responses in investigating the acute impact of nutritional interventions on cognitive performance and mood states, with a particular focus on stress and alertness, in healthy adult populations. This approach shows great promise in enhancing the accuracy and validity of research findings in this field. Notably, it is supported by theories that link ANS responses with mood and cognitive function [12,14,57]. Furthermore, the use of ANS responses provides several experimental advantages, including real-time and objective measures [9,58].

Our review sheds light on the potential of ANS responses to enhance our understanding of the acute effects of nutrition interventions on cognitive performance, stress, and alertness [15,16,17,18,19,20,21,22,23,24,25,26,27,28,37]. By continuously monitoring ANS responses, researchers can track and evaluate the temporal dynamics of dietary components’ effects, whether they are due to sensorial stimulation or a bioactive mechanism. ANS responses may change immediately due to direct stimulation of the senses, while post-absorption changes in ANS data may reflect variations in response to the mechanism of the bioactive compound being tested [27]. It is important to note that changes in ANS responses, when testing food or beverage matrices, may result from interactions between matrix constituents [27,46]. A subjective rating can then generate evidence to support objective proofs provided by ANS responses on any change in alertness or stress induced by the dietary component [19,27]. Similarly, the performance of a reaction time task can complement SNS responses to demonstrate the potential of an acute nutritional intervention to enhance alertness [33].

Intervention studies that have evaluated the effects of dietary components on stress, alertness, and cognitive performance using ANS responses as outcome measures have reported inconsistent results [16,44,46,50]. The inconsistency in findings could be due to the complex relationship between ANS responses, mood states, and cognitive performance. One factor contributing to this complexity is that different mood states sharing similar valence and arousal can lead to similar ANS responses [59]. Moreover, individual differences, such as daily consumption of the tested component or age, body weight and sex, can significantly complicate the relationship between ANS responses, mood states, and cognitive function [53,54,60]. Another challenge is achieving comprehensive results across ANS responses, cognitive tasks, and subjective ratings, as they reflect different aspects of an individual’s physiological and psychological states [61]. Specifically, the central nervous circuitry appears to be the primary driver of cognitive changes, potentially explaining the disconnection between ANS responses and cognitive performance observed in some studies [35,50]. Other physiological systems might mediate the modulation of ANS responses in acute nutritional interventions e.g., the baroreflex-mediated response in caffeine interventions that impact ANS responses would confound changes associated with the alertness state [27,44]. While the experimental setup associated with the reviewed approach provides the convenience of real-time and continuous measurement, it might not reflect naturalistic effects since it requires participants to remain seated and motionless in front of a computer [62]. Future studies should take advantage of recent developments in wearable and portable devices to test the impact of dietary components in a real-world setting rather than a laboratory environment in which the participant can focus their attention on the experimental setup [63]. It is worth noting that this attention bias might have influenced the ECG-based testing of the effect of chewing gum in the study conducted by Allen AP, Jacob TJ and Smith AP [51], given the participants’ high baseline subjective alertness. Real-world and real-time observations offer the chance to examine the ecological validity and robustness of existing health claims [64], as well as to substantiate new diet–health associations through the use of a large variety of study designs and intensive longitudinal data collection [65]. Such an approach would enable us to capture (1) patterns in changes over time, (2) individual responses to nutritional interventions as these unfold over time, and (3) time-dependent and (4) context-dependent responses. However, the current issues faced by the easy-access technologies, including the high vulnerability to motion artifacts and the lack of access to proprietary algorithms, limit their applications in nutrition studies.

Overall, ANS responses are reliable and valid objective measures that can be used to explore the impact of acute nutritional interventions on mood states and cognitive function. Approaches to developing both hardware as well as software solutions are ongoing. Algorithms for automatic detection of mood and cognitive changes might be integrated to multi-sensor platforms, wearables, or contactless devices to assess food or beverage effects as they occur in peoples’ daily environments. The combination of current advances in wearable devices and digital technologies has made it possible to monitor nutrition and physiology in real-world, real-time settings and conduct large-scale studies with repeated observations and collection of big data. These developments, along with the use of machine learning algorithms, open up new possibilities for personalized nutritional interventions and recommendations [65]. Tailoring nutritional advice, products or services to individual variability, characteristics and needs represents the main goal of the growing field of personalized nutrition [66]. Compared with generalized approaches, personalized nutrition may lead to significant enhancement of health outcomes as well as behavioral changes [67]. This is achieved through the use of external monitoring devices, which inform individuals about their physiological state and effectively facilitate the adoption of healthy behaviors by boosting motivation and precisely targeting desired health outcomes [68]. However, this approach requires richer and more robust data to account for variables inherent to ecological setup and further research is needed to better validate the link between ANS responses, mood states, and cognitive function.

## Figures and Tables

**Table 1:** Summary of studies testing the acute (within a day) effects of dietary components on stress using ANS responses as outcome measures in healthy adult populations.

Reference	Design	Interventions	Population	Key Endpoints	Key Results
Zahar S, Schneider N, Makwana A, Chapman S, Corthesy J, Amico M and Hudry J [35]	Randomized, double-blind, 3-arm parallel, placebo-controlled	0.5 or 1 g tryptophan in an egg protein hydrolysate	45 healthy subjects (mean age 33.3 ± 6.15 SD yo)	- Self-report of stress state (VAS) - Cognitive performance (RVIP) - Physiological measures (HRV and GSR)	Tryptophan interventions versus placebo: Higher HRV during a multi-task stressor
Kimura K, Ozeki M, Juneja LR and Ohira H [36]	Randomized, double-blind, 2-arm cross-over, placebo-controlled	200 mg L-theanine	12 male undergraduate students (mean age 21.50 ± 1.38 SD yo)	- Self-report of stress state (VAS and STAI) - Physiological measure (HRV)	L-theanine intervention versus placebo: Lower HR during a task stressor
Kennedy DO, Bonnlander B, Lang SC, Pischel I, Forster J, Khan J, Jackson PA and Wightman EL [16]	Randomized, double-blind, 4-arm parallel, placebo-controlled	430 mg, 860 mg, or 1290 mg green oat extract	132 healthy subjects (age range 35–65 yo)	- Self-report of alertness and calmness state (Bond-Lader Mood Scales) - Cognitive performance (Numeric Working Memory, Corsi Blocks, RVIP, Stroop, serial subtraction and tracking) - Physiological measures (HR and GSR)	Green oat intervention (860 mg) versus placebo: Higher HR during an observed multi-task stressor
Boyle NB, Billington J, Lawton C, Quadt F and Dye L [38]	Randomized, double-blind, 4-arm parallel, placebo-controlled	Mg + B vitamins + green tea + rhodiola extract, Mg + B vitamins + rhodiola extract or Mg + B vitamins + green tea -Mg: 150 mg elemental -Green tea: 125 mg containing 40% L-Theanine -Rhodiola extract: 222 mg -B vitamins: 0.7, 0.1, and 0.00125 mg of vitamins $B_{6}$, $B_{9}$ and $B_{12}$, respectively	100 healthy subjects (age range 18–50 yo)	- Self-report of alertness (Bond-Lader VAS) and stress (DASS, SACL) states - Physiological measure (HRV)	Intervention of Mg + B vitamins + green tea + rhodiola extract versus a placebo: Lower SACL stress score & HRV during a social stress test
Eckstein ML, Brockfeld A, Haupt S, Schierbauer JR, Zimmer RT, Wachsmuth NB, Zunner BEM, Zimmermann P, Erlmann M and Obermayer-Pietsch B [17]	Randomized, double-blind, 4-arm cross-over, placebo-controlled	1 g/kg BM Glucose, 1 g/kg BM Fructose, or 0.5 g/kg BM of Glucose + Fructose	15 healthy subjects (age range 18–65 yo)	- Physiological measure (HRV)	Carbohydrate interventions versus a placebo: Lower HRV
Jung D-J, Cha J-Y, Kim S-E, Ko I-G and Jee Y-S [37]	2-arm parallel, placebo-controlled	20-min inhalation of Ylang-Ylang essential oil (Cananga odorata)	15 healthy males in the Ylang-Ylang essential oil group (mean age 21.07 ± 0.43 SEM yo) 14 males in placebo group (mean age 22.00 ± 0.58 SEM yo).	- Physiological measure (HR)	Ylang-Ylang essential oil intervention versus a placebo: Lower HR

BM: Body Mass, DASS: Depression Anxiety and Stress Scale, GSR: Galvanic Skin Response, HR: Heart Rate, HRV: Heart Rate Variability, Mg: Magnesium, RVIP: Rapid Visual Information Processing, SACL: Stress and Arousal Checklist, STAI: State-Trait Anxiety Inventory, SD: Standard Deviation, SEM: Standard Error Mean, VAS: Visual Analog Scale, yo: years old.

**Table 2:** Summary of studies testing the acute (within a day) effects of dietary components on alertness using ANS responses as outcome measures in healthy adult populations.

Reference	Design	Interventions	Population	Key Endpoints	Key Results
Redondo B, Vera J, Carreño--Rodríguez C, Molina-Romero R and Jiménez R [19]	Double-blind, 2-arm cross-over, placebo-controlled	4 mg of caffeine per 1 kg of body weight	22 university students (21.68 ± 3.67 SD yo)	- Self-report of alertness state (Stanford Sleepiness Scale) - Physiological measures (pupil diameter)	Caffeine intervention versus a placebo: Higher pupil dilation & alertness score
Quinlan PT, Lane J, Moore KL, Aspen J, Rycroft JA and O’Brien DC [26]—Study 1	Randomized, 5-arm cross-over, placebo-controlled	Hot water, tea (37.5 mg caffeine), tea (75 mg caffeine), or coffee (75 or 150 mg)	17 healthy subjects (age range 21–51 yo)	- Self-report of alertness state (LARS) - Physiological measures (HR, GSR, skin temperature)	Caffeine interventions versus hot water: Higher LARS alertness scores (with a dose-related increase) Caffeine and hot water interventions versus a placebo: Higher HR, GSR & skin temperature
Quinlan PT, Lane J, Moore KL, Aspen J, Rycroft JA and O’Brien DC [26]—Study 2	Randomized, double-blind, 5-arm cross-over, placebo-controlled	Hot water, decaffeinated (~5 mg caffeine), or decaffeinated tea with caffeine (30, 55, 105, or 205 mg caffeine)	17 healthy subjects (age range 22–52 yo)	- Self-report of arousal and valence (UWIST) - Physiological measures (HR, GSR and skin temperature)	Caffeine interventions versus hot water: Modulation of arousal (with a dose-related U-shaped pattern), lower HR & skin temperature (with a dose-related decrease) and higher GSR Hot water versus placebo: Higher skin temperature, GSR & HR
Schneider R [33]	Randomized, double-blind, 4-arm cross-over, placebo-controlled	100% natural essential oils from peppermint, rosemary, and grapefruit (ratio: 50-30-20), 100% natural peppermint, rosemary, and cinnamon oil (ratio: 86-11-3), or 250 mL of commercialized energy drink	50 health subjects (mean age 34.2 ± 6.9 SD yo)	- Cognitive performance (CompACT-Vi, mental arithmetic) - Physiological measure (HR)	Essential oil versus an energy drink intervention or a placebo: Higher HRV & RT

CompACT-Vi: Comprehensive Computerized Attention and Cognitive Concentration Tests-Vigilance, GSR: Galvanic Skin Response, HR: Heart Rate, LARS: Line Analogue Ratings Scale, RT: Reaction Time, SD: Standard Deviation, UWIST: University of Wales Institute of Science & Technology, yo: years old.

**Table 3:** Summary of studies testing the acute (within a day) effects of dietary components on cognitive performance using ANS responses as outcome measures in healthy adult populations.

Reference	Design	Interventions	Population	Key Endpoints	Key Results
Bichler A, Swenson A and Harris M [44]	Randomized, double-blind, 2-arm cross-over, placebo-controlled	100 mg caffeine + 1000 mg taurine	14 undergraduate students (mean age 20.5 ± 1.5 SD yo)	- Cognitive performance (Experimental Comparative Prediction Battery) - Physiological measure (HR)	Caffeine + taurine versus placebo: Lower HR
Scholey AB and Kennedy DO [45]	Randomized, double-blind, 5-arm cross-over, placebo-controlled	250 mL water with 75 mg caffeine, 37.5 g glucose, herbal flavoring fractions, or 75 mg caffeine, 37.5 g glucose, and herbal flavoring fractions.	20 undergraduate volunteers (age range 18–32 yo)	- Self-report of alertness state (Bond-lader scale) - Cognitive performance (DSST, CDR) - Physiological measure (HR)	Glucose intervention versus placebo: Higher HR Caffeine intervention versus placebo: Lower HR Glucose + flavoring fractions intervention versus placebo: Higher cognitive performance
Allen AP and Smith AP [50]—Study 4	2-arm, cross-over, placebo-controlled	1 full packet of chewing gum	30 full-time university staff (mean age 30.4 ± 6.9 SD yo)	- Self-reports on alertness state and cognitive performance (Unpublished questionnaires) - Physiological measure (HR)	Chewing gum intervention versus placebo: Respectively higher and lower scores associated with the work done and the cognitive problems during a workday
Allen AP, Jacob TJ and Smith AP [51]	Randomized, parallel, 2-arm, placebo-controlled	Gum (gum base, glycerine, lecithin, sorbitol, emulsifier and sweeteners including aspartame and acesulfame K)	Gum condition: 18 participants (mean age 22.33 ± 2.5 SD yo) Control: 24 participants (mean age 24.4 ± 3 SD yo)	- Self-report of alertness (VAS) - Cognitive performance (repeated digits) - Physiological measure (HR)	Chewing gum intervention versus placebo: Higher Hits and, lower RT &HR
Pomportes et al. [52]	Randomized, double-blind, 3-arm, cross-over, placebo-controlled	A supplement with vitamin, mineral and, 300 mg guarana supplement or a 100 mg caffeine supplement	56 participants (males age range 19–45 yo; females age range 18–42 yo)	- Cognitive performance (Go-no go; SRT) - Physiological measure (HR)	Vitamin + mineral + guarana intervention versus a placebo: Lower RT Caffeine intervention and placebo: Decrease of HRV

CDR: Cognitive Drug Research, DSST: Digit Symbol Substitution Task, HR: Heart Rate, HRV: Heart Rate Variability, SD: Standard Deviation, SRT: Simple Reaction Time, yo: years old.

## Data Availability

No new data was created or analyzed in this work. Data sharing is not applicable to this article.
